# Lentivirus Display: Stable Expression of Human Antibodies on the Surface of Human Cells and Virus Particles

**DOI:** 10.1371/journal.pone.0003181

**Published:** 2008-09-11

**Authors:** Ran Taube, Quan Zhu, Chen Xu, Felipe Diaz-Griffero, Jianhua Sui, Erick Kamau, Markryan Dwyer, Daniel Aird, Wayne A. Marasco

**Affiliations:** 1 Department of Cancer Immunology and AIDS, Dana-Farber Cancer Institute, Boston, Massachusetts, United States of America; 2 Department of Medicine, Harvard Medical School, Boston, Massachusetts, United States of America; 3 Department of Pathology, Harvard Medical School, Boston, Massachusetts, United States of America; University of Arkansas for Medical Sciences, United States of America

## Abstract

**Background:**

Isolation of human antibodies using current display technologies can be limited by constraints on protein expression, folding and post-translational modifications. Here we describe a discovery platform that utilizes self-inactivating (SIN) lentiviral vectors for the surface display of high-affinity single-chain variable region (scFv) antibody fragments on human cells and lentivirus particles.

**Methodology/Principal Findings:**

Bivalent scFvFc human antibodies were fused in frame with different transmembrane (TM) anchoring moieties to allow efficient high-level expression on human cells and the optimal TM was identified. The addition of an eight amino acid HIV-1 gp41 envelope incorporation motif further increased scFvFc expression on human cells and incorporation into lentiviral particles. Both antibody-displaying human cells and virus particles bound antigen specifically. Sulfation of CDR tyrosine residues, a property recently shown to broaden antibody binding affinity and antigen recognition was also demonstrated. High level scFvFc expression and stable integration was achieved in human cells following transduction with IRES containing bicistronic SIN lentivectors encoding ZsGreen when scFvFc fusion proteins were expressed from the first cassette. Up to 10^6^-fold enrichment of antibody expressing cells was achieved with one round of antigen coupled magnetic bead pre-selection followed by FACS sorting. Finally, the scFvFc displaying human cells could be used directly in functional biological screens with remarkable sensitivity.

**Conclusions/Significance:**

This antibody display platform will complement existing technologies by virtue of providing properties unique to lentiviruses and antibody expression in human cells, which, in turn, may aid the discovery of novel therapeutic human mAbs.

## Introduction

Monoclonal antibodies (mAbs) have been used with increasing frequency to treat a wide spectrum of human diseases, including heart disease, infections and immune disorders [1]–[5]. The mAb based immunotherapies are now standard of care in an increasing number of human cancers including Erb2+ breast cancer, Non-Hodgkin's Lymphoma, colon cancer and others [1], [6], [7].

Since 2001, human mAbs developed through recombinant DNA techniques have constituted the largest number entering clinical study [1]. This shift, toward *de novo* human mAb isolation and their clinical use, is in part due to new antibody display and other library screening techniques, which are now being exploited to isolate human antibodies with high affinity and specificity. The microbial surface display technologies for screening antibody libraries include phage, yeast and bacteria. Phage-display is widely used due to its simplicity, versatility and ability to be adapted to many specific conditions, including selection on whole cells and tissues [8]. Yeast and bacteria display platforms have several advantages over the phage system including use of flow-cytometry and sorting techniques to enable finer affinity discrimination of selected antibodies [9], [10]. Among the non-microbial systems is ribosomal display that has the capacity to screen libraries of greater size as well as facilitating diversity and efficient antibody maturation *in vitro*.

Although isolation of human antibodies from the above mentioned systems has been successful, there can be unexpected problems with subsequent therapeutic mAb development due to constraints of protein expression, correct folding and post-translational modifications. This has been particularly true for antibodies isolated by phage-display technology. There has been great interest in screening antibodies directly from mammalian cells due to their ability to provide proper posttranslational modification, as well as the existence of the natural chaperones that assist in antibody folding. Animal cells have been used for the direct screening of hypermutating antibodies [11], [12] and during antibody selection from a retroviral-antibody display library [13]. Transient antibody expression on the surface of human 293T has also been recently reported as a system to perform *in vitro* affinity maturation of human antibodies [14]. Furthermore, sulfation of tyrosine residues in the CDR residues of human antibodies can markedly affect antigen recognition [15], [16] and contribute bidirectionally to the binding activity of antibodies [17]. These latter findings suggest that antibody selection and expression on the surface of human cells may not only identify a population of antibodies that would be difficult or even impossible to detect in other microbial or cell-free display systems, which lack the ability to sulfonate CDR tyrosines, but may also be able to select against antibodies that may otherwise loose activity upon transferring to mammalian expression systems.

In this report, we show that bivalent functional human scFvFc fusion proteins can be efficiently expressed on surface of lentiviral transduced human cells, as well as incorporated onto the surface of lentiviral particles. The displayed scFvFc antibodies can undergo post-translational CDR tyrosine sulfation. Combined magnetic bead and FACS selections on transduced human cells have provided, proof-in-principle, that 10^6^-fold enrichments of specific antibodies can be achieved in a single, rapid selection step. In addition, scFvFc displaying human cells could be used directly in functional biological screens with remarkable sensitivity.

## Results

### Optimization of scFv surface expression in mammalian cells

**Figure 1:**
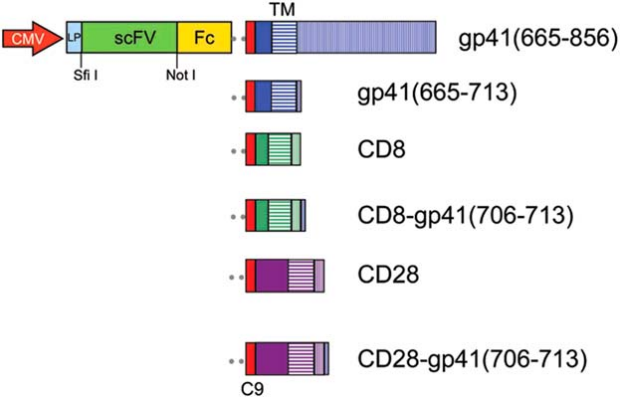
Diagram of constructs used in the study. ScFv antibodies were inserted between the leader peptide (LP) and the Fc region of a human IgG1 molecule. The Fc domain was linked in-frame to a short segment of extracellular domain of HIV-1 gp41 (blue), CD8 (green) or CD28 (purple), followed by their respective transmembrane domains (TM; horizontal stripes) and cytoplasmic domains (vertical stripes). In the case of HIV-gp41, the last 19 residues of the extracellular region (solid blue) are followed by a TM spanner (22 residues; blue horizontal stripes) and a cytoplasmic tail (blue vertical stripes). Either the full length 151 residues of the cytoplasmic domain or a truncated region that includes only the first eight residues of the cytoplasmic tail were used. Numbering is according to p160 of HIV-1 HXB2. For CD8, the most membrane-proximal 12 residues of the extracellular domain (solid green) and 11 residues of the cytoplasmic domain (green vertical stripes) flank 21 residues of the TM region (horizontal green stripes). For CD28, an extracellular region consisting of 40 residues (solid purple) and a cytoplasmic region of 13 residues (purple vertical stripes) flank the 27-residue TM domain (horizontal purple stripes). To facilitate scFvFc-TM incorporation into virions, an eight-residue “*env* incorporation motif”, which encodes the membrane proximal part of the gp41 cytoplasmic tail (NRVRQGYS; single blue line-amino acids 706–713), was attached to the carboxy-terminal ends of the cytoplasmic domains of CD8 and CD28. A nine amino acid C9 tag (red box) is positioned at C-terminus of all Fc domains to facilitate detection/quantitation of scFvFc expression on the cell surface. The gene cassette was cloned into pCDNA3.1 or the modified pHAGE lentiviral vector between Sfi-I and Pac-I sites. A CMV promoter controls expression of the scFvFc-TM transgenes.

PS11 scFv, an antibody targeting the Tat-recognition motif (TRM) of cyclin T1 [18], was chosen as a model for optimizing functional expression of scFv on the surface of mammalian cells. To gain bivalency and increase the sensitivity of detecting antigens bound to surface antibody, the PS11 scFv was expressed as an scFvFc fusion protein [18]–[20]. For anchoring to the cell membrane, PS11 scFvFc protein was fused, in frame, to a transmembrane (TM) moiety. TM domains of HIV-1 gp41, CD8 and CD28 were tested for maximal surface expression of the scFvFc. As shown in Figure 1, all anchoring moieties consist of a short extracellular region, an entire TM domain and a cytoplasmic tail. Eight residues of the most membrane-proximal HIV-1 gp41 cytoplasmic tail, previously shown to provide a putative “envelope (*env*) incorporation motif” [21], were also tested for their ability to promote efficient pseudotyping of the scFvFc fusion proteins onto HIV virions or subsequently the cell surface, as a direct fusion to the gp41 TM or attached to the carboxy terminus of the CD8 or CD28 cytoplasmic tails.

**Figure 2:**
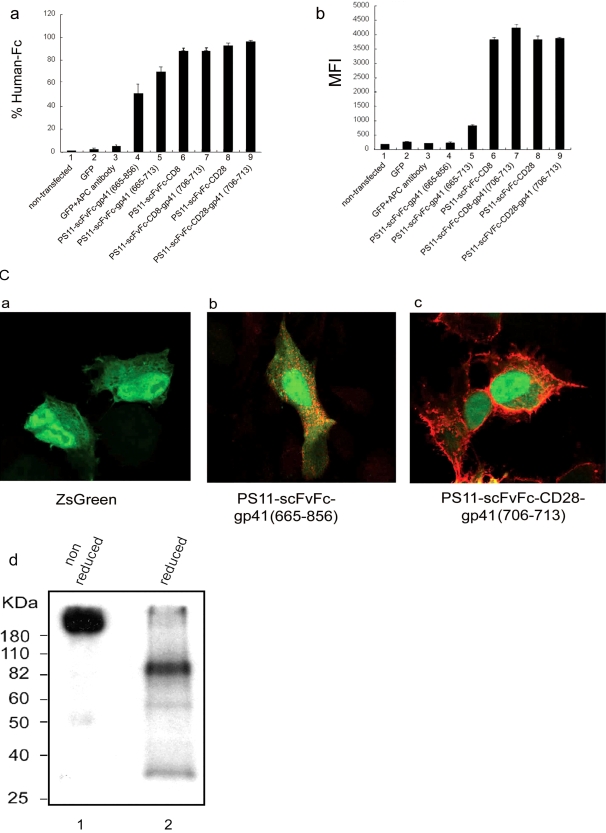
Optimization of scFvFc cell-surface expression using different transmembrane domains. 293T cells were transfected with the pcDNA 3.1 based constructs encoding PS11-scFvFc antibodies of different configurations as described in Figure 1 and labeled under each lane in Panels a and b. pcDNA3.1-CMV-GFP was co-transfected as an internal control for transfection efficiency. At 48 hours post transfection, cells were harvested and analyzed for GFP and scFv-Fc expression by FACS analysis. *Panels a* and *b*, represent results from FACS analysis of the percentage of cells that are positive for APC-anti-human Fc staining (*a*) and their respective MFI values (*b*). Error bars represent the standard deviation of the average of three experiments. *Panel c*. Cellular localization of the PS11-scFvFc-TM analyzed by confocal immunomicroscopy. 293T cells were transfected with either ZsGreen expression vector alone, or with a bicistronic vector expressing both the PS11 scFvFc-TM fusion proteins and ZsGreen. At 48 hours post transfection, cells were stained with a rhodamine-conjugated anti-human Fc for the detection of scFvFc expression as visualized by a confocal microscope. *Image a*, cells transfected with ZsGreen only vector; *Images b* *and* *c*, cells transfected with vectors expressing either PS11-scFvFc-gp41 (665–856)-IRES ZsGreen or PS11-scFvFc-CD28-gp41 (706–713)-IRES-ZsGreen, respectively. Absence of the ZsGreen fluorescence in some of the APC+ cells is likely the result of low level expression of ZsGreen from the second cassette of the bi-cistronic message. *Panel d*. PS11-scFv-CD28-gp41 is present as a dimer in transfected cells. 293T cells expressing pCDNA3.1-PS11-scFvFc-CD28-gp41 fusion protein were metabolically labeled with [^35^S]-cysteine and [^35^S]-methionine mixture. Cell lysates were immunoprecipitated with protein A sepharose beads, resuspended with 2× SDS non-reducing (lane 1) or reducing buffer (lane 2), and subjected to SDS-PAGE and autoradiogram.

Surface expression of the PS11-scFvFc-TM proteins was initially analyzed by FACS analysis of transiently transfected 293T cells, stained with APC-conjugated anti-human-Fc antibody (Figure 2). While transfection efficiency with each of the constructs was relatively equal, as monitored through the expression level of a co-transfected GFP plasmid (data not shown), depending on the transmembrane moiety, differences in cell-surface expression of PS11-scFvFc were observed in both the percentage of cells that were positive for scFvFc expression (Panel a), and more pronounced by their respective MFI values (Panel b). The data indicate that PS11-scFvFc antibodies anchored by the TM of CD8 (lanes 6 and 7) or CD28 (lanes 8 and 9) were highly expressed on the surface of mammalian cells, compared to PS11-scFvFc fused to HIV-gp41 TM (either a long or short cytoplasmic tails; lanes 4 and 5) that were poorly surface expressed, and their MFI values were low.

To directly visualize the distribution and localization of scFvFc-TM expression, cells transfected with a bicistronic IRES-ZsGreen expression vector encoding PS11-scFvFc-gp41 (665–856), or PS11-scFvFc-CD28-gp41 (706–713) were labeled with a rhodamine conjugated anti-human Fc antibody for immunofluorescence analysis. As shown in Figure 2c, cells expressing PS11-scFvFc-gp41 (665–856) demonstrated punctate staining with large aggregates and exhibited an overall low level of cell-surface expression (image b). In contrast, PS11-scFvFc-CD28-gp41 (706–713) proteins were evenly distributed on the cell surface and also had a reticular staining pattern, consistent with efficient ER folding and expression (image c). As a control, rhodamine conjugated anti-human Fc staining was not detected on cells transfected with ZsGreen encoding vector alone (image a). These results are consistent with the FACS data shown in Figure 2a and 2b. Low expression and possible aggregation of PS11-scFvFc-gp41 (665–856) may be a result of poor folding, as the natural Fc moiety forms dimers, while gp41 forms trimers through its TM. Finally, radio-immunoprecipitation and SDS-PAGE analysis confirmed that the membrane bound PS11-scFv-CD28-gp41 (706–713) protein was dimeric (Figure 2d).

### Specific antigen binding by mammalian cell-surface expressed scFvFc-TM antibodies

**Figure 3:**
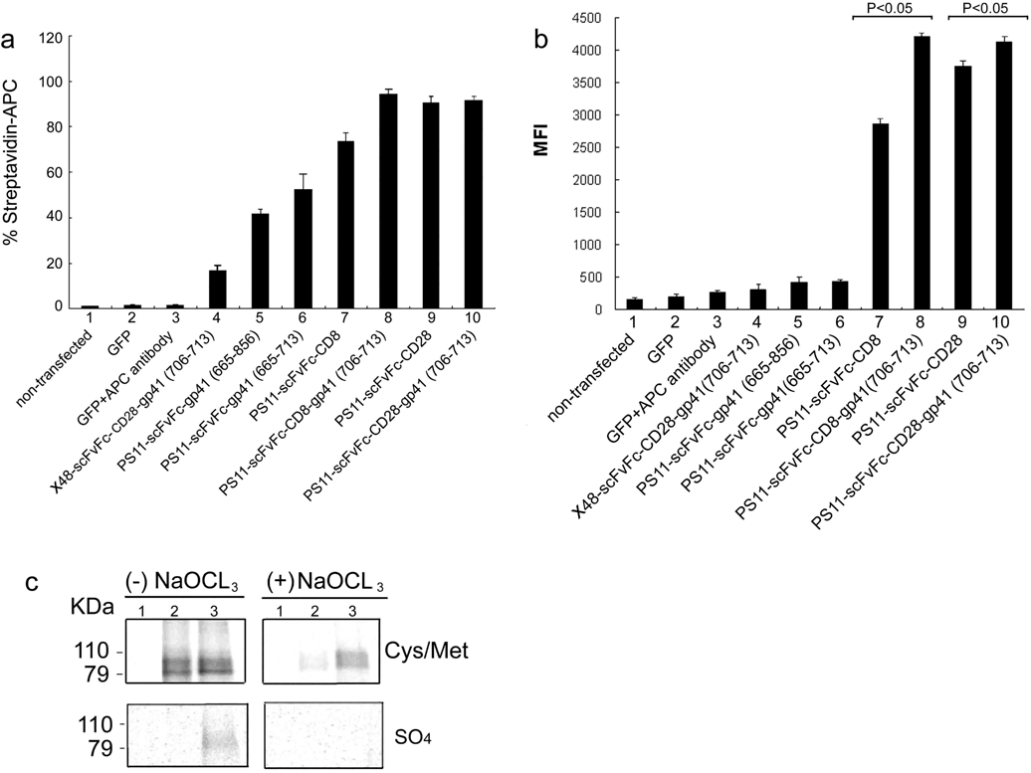
Cell surface expressed scFvFc proteins bind their cognate antigens. 293T cells were transfected with the same constructs as described in Figure 2 and labeled under each lane in Panels a & b. Two additional constructs encoding antibodies against CXCR4, X20- and X48-scFvFc-CD28-gp41, were also transfected. pcDNA3.1-CMV-GFP was again co-transfected as an internal control for transfection efficiency. At 48 hours post transfection, cells were harvested and stained for biotinylated-TRM and streptavidin-APC, followed by FACS analysis. GFP expression was also analyzed to ensure equal transfection efficiencies. *Panel a* and *b*, depict the percentage of positive cells that express a functional PS11 scFvFc as determined by staining with streptavidin-APC (Panel a) and their respective MFI values (Panel b). Error bars represent the standard deviation of the average of three experiments. P values<0.05 above the designated bars, represent statistically significant difference in MFI values. *Panel c.* Post-translational sulfation occurs in selected surface displayed scFvFc antibodies. 293T cells expressing cell surface X48 or X20-scFcFc-CD28-gp41 fusion proteins (lanes 2 and 3, respectively) were labeled with [^35^S]-cysteine and [^35^S]-methionine mixture (upper panel; Cys/Met) or with [^35^S]-sulfate (lower panel; SO_4_) with or without 100 mM sodium chlorate treatment. Cell lysates were immunoprecipitated with protein A sepharose beads, washed and analyzed by SDS-PAGE and autoradiography. pcDNA3.1 backbone empty vector was also used as negative control (lane 1).

To confirm that the cell surface-anchored PS11-scFvFc remains functional for binding to its antigen, 293T cells transfected with different PS11-scFvFc-TM fusion proteins were stained with a biotinylated-TRM peptide and analyzed by FACS, using APC-conjugated streptavidin. As seen in Figure 3, all PS11-scFvFc-TM proteins were functional for binding the biotinylated TRM peptide. The binding was specific, since an irrelevant X48-scFvFc, recognizing a biotinylated CXCR4 peptide [17], showed only a relatively low level of APC-streptavidin staining (lane 4). Overall, the PS11-scFvFc-CD8-gp41 and PS11-scFvFc-CD28-gp41 proteins were the most competent in binding the peptide and exhibited higher levels of peptide binding compared to the corresponding constructs without the envelope incorporation motifs (Figure 3b, compare lanes 8 and 10 to lanes 7 and 9, respectively). This result suggests that this motif may stabilize surface antibody expression, by promoting its proper folding and membrane association. The lower surface expression of PS11-scFvFc linked to the gp41 TM region (Figure 2a and b) lead to a dramatically lower binding capacity to the biotinylated TRM peptide, as compared with the PS11-scFvFc fused to the CD8 or CD28 TM regions, evidenced by both a lower percentage of antigen binding cells and MFI values (Figure 3a and b; compare lanes 5 and 6 with lanes 7 and 9).

### Tyrosine sulfation of the mammalian cell-surface expressed scFvFc-CD28-gp41 antibodies

We have demonstrated that in self-reactive human anti-CXCR4 antibodies, tyrosine sulfation occurs in novel areas of the V-region genes and contributes bidirectionally to antibody binding activity [17]. To determine if tyrosine sulfation could also occur on surface displayed scFvFc, the self-reactive human anti-CXCR4 antibodies X20- and X48-scFv were analyzed as scFvFc-CD28-gp41 (706–713) fusion proteins. Radioimmunoprecipitation studies confirmed that sulfation indeed occurred in the surface displayed X20-scFvFc-CD28-gp41 but not with X48-scFvFc-CD28-gp41 (Figure 3c, compare lower lanes 3 and 2, respectively). Treatment of transfected cells with sodium-chlorate, a sulfation inhibitor [15], [22], decreased expression but more significantly abolished sulfation of scFvFc proteins (Figure 3c lower and upper panels, respectively). This is consistent with the results for each corresponding soluble scFv, where sulfation was mapped to tyrosine in VH CDR2 and VL FW3 regions of X20 and required for maximal binding and antigen recognition activity [17].

### Incorporation of functional scFvFc-TM proteins into lentivirus particles

The IgG leader-scFvFc-CD28/CD28-gp41 (706–713) coding sequences were next cloned into the first cassette of a bicistronic self-inactivating (SIN) lentivector containing an IRES-ZsGreen reporter gene. Viruses encoding PS11-scFvFc, X48-scFvFc, as well as two SARS-CoV specific antibodies 80R-scFvFc (19, 23) and 11A-scFvFc (Sui et al, submitted) that recognize Tor2 or GD03 Spike protein, respectively, were produced through co-transfecting cells with HIV packaging plasmid and a VSV-G envelope DNA plasmid providing surface binding and fusogenic activity for viral entry. These viruses were analyzed for incorporation of scFvFc into the viral envelope and their capacity to bind specific antigens; and were further used to establish a mammalian cell display of surface-bound antibodies through transduction.

**Figure 4:**
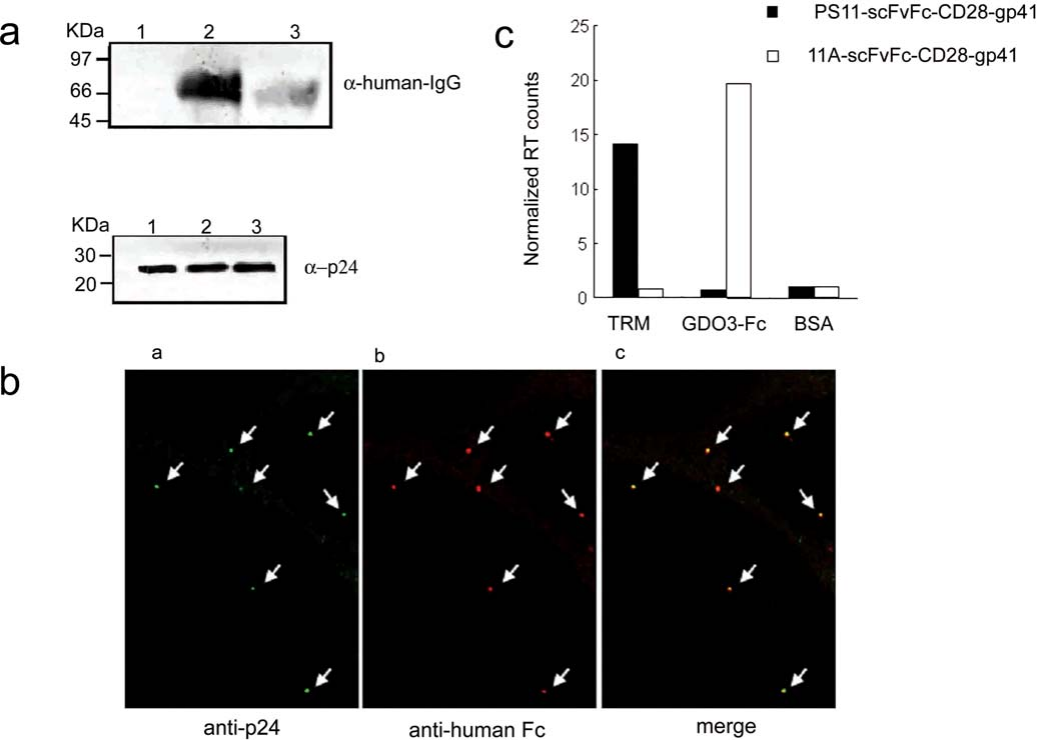
Functional scFvFc are incorporated into lentivirus particles. *Panel a.* An HIV-1 gp41 incorporation motif enhances scFvFc incorporation into viral particles-equal loads of lentiviruses encoding ZsGreen (lane 1), PS11-scFvFc-CD28-gp41-IRES ZsGreen (lane 2) or PS11-scFvFc-CD28-IRES ZsGreen (lane 3) were subjected to SDS-PAGE analysis, followed by western blotting using either HRP-conjugated anti-human Fc (upper panel) or anti-HIV-1 p24 (lower panel) antibodies. *Panel b.* Immunostaining of viruses. Viral particles were attached to Hela cells for 2 hour at 4°C. Cells were then fixed and stained with anti-HIV p24 antibody followed by Cy2-conjugated anti-mouse IgG or a rhodamine-conjugated anti-human Fc for surface Fc staining. Following these procedures, cells were washed and analyzed by confocal microscope. Shown separately are viruses stained for detection of p24 (image a) and scFvFc (image b) along with a merged image (image c). *Panel c.* Antigen specific capture of lentiviruses displaying corresponding scFvFc antibodies. Equal amounts of lentivirus particles expressing on their surface either the PS11-scFvFc or the 11A-scFvFc were loaded on a 96-well plate that was coated with the following specific antigens: TRM-peptide (PS11 specific), GD03-Fc (11A specific) or BSA. Following incubation to allow capture of the viruses to the antigens, wells were washed extensively and viral particles were eluted and quantitated by RT assay. Presented are normalized RT counts, where RT counts of particles bound to their antigens were divided by the RT counts of virus bound to the BSA control. Values are the average of duplicated samples and data are representative of two separate experiments.

Incorporation of scFvFc-TM was first examined by western blot analysis of equal amounts of viral particles, as determined by p24 levels (Figure 4a, lower panel). Using an anti-human Fc antibody, both PS11-scFvFc-CD28-gp41 (706–713) and PS11-scFvFc-CD28 were detected in the purified viral particles (Figure 4a, upper panel, lanes 2 and 3, respectively), while a control CMV-GFP lentivirus showed no reactivity (upper lane 1). Most importantly, the gp41 (706–713) *env* incorporation motif-encoding viruses exhibited higher Fc expression (compare upper lanes 2 and 3), confirming a more efficient incorporation of the PS11-scFvFc-CD28-gp41 into lentiviral particles, which is in agreement with the results obtained in Figure 3b.

Upon binding and fixation of purified viral particles to Hela cells, an immunostaining protocol was performed to confirm that viral particles incorporated the scFvFc fusion proteins. Confocal microscopic images shown in Figure 4b indicated that viruses expressing PS11-scFvFc-CD28-gp41 (706–713) on their surface were stained with both anti-HIV-1 p24 antibody (image a) and anti-human Fc antibody (image b). Merging of the two staining profiles confirmed co-localization of the core with scFvFc (image c). Control CMV-GFP viral particles, with no scFvFc molecules on their surface, stained positively with the anti-p24 antibody only (data not shown).

To further verify that scFvFc-CD28-gp41 (706–713) proteins are displayed on the surface of lentivirus and remain functional, a virion capture experiment was performed. Equal amounts of viral particles [based on reverse transcription (RT) value], expressing on their surface either PS11- or 11A-scFvFcs, were incubated in a 96-well plate where wells were coated with either biotinylated TRM peptide (PS11-scFvFc specific) or GD03-Fc protein (11A-scFvFc specific) antigen. BSA served as a negative control. Following binding and extensive washing, the amount of captured viral particles in each well was determined by RT assay. As shown in Figure 4c, each virus bound to its own target with a very high selectivity/specificity. Hence, recombinant lentiviral particles could be efficiently pseudotyped with functionally intact scFvFc-CD28-gp41 fusion proteins.

### Characterization of scFvsFc expressed on the surface of lentivirus transduced cells

**Figure 5:**
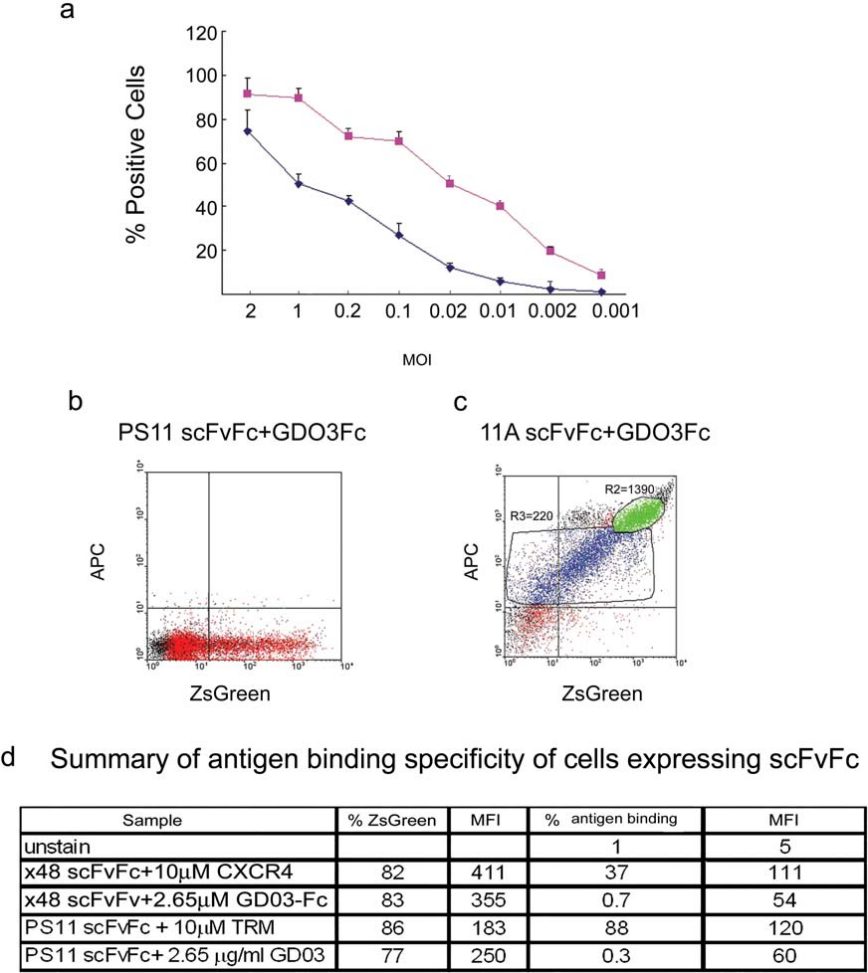
Expression of scFvFc on the surface of lentivirus transduced cells. *Panel a* 293T cells were transduced with increasing dilutions (different MOIs as indicated) of lentivirus encoding the PS11-scFvFc-CD28-gp41-IRES-ZsGreen. Transduced cells were harvested, stained for Fc-surface expression, and analyzed by FACS. Expression of ZsGreen was measured to monitor levels of transduction. The graphs depict the percentage of transduced cells that express ZsGreen (blue diamonds) and the percentage values of transduced cells that express PS11-scFvFc as monitored by staining with APC-conjugated anti-human Fc IgG (pink squares). *Panels b* and *c*, cell -surface expressed scFvFc proteins are functional. 293T cells were transduced with a lentivirus encoding PS11-scFv-Fc-CD28-gp41-IRES-ZsGreen (*Panel b*) or 11A-scFvFc-CD28-gp41-IRES-ZsGreen (*Panel c*). Cells were incubated with biotinylated GD03-Fc, a specific antigen for 11A-scFvFc, and stained for streptavidin-APC as described in Methods, and then analyzed by FACS. Note that two clusters of cells in *Panel c* represent high (R2 gated) and low (R3 gated) levels of 11A-scFvFc on their surface as measured by APC staining. These could reflect variations in cell-surface expression levels resulting from multiple integration events of the scFvFc cassette following transduction, or the difference in transgene integration site, *i.e.*, its proximity to active transcriptional units. R2 = 1390 and R3 = 220 are MFI values of 11A scFvFc expressing cells, where percentage of positive cells in each gate is 31% and 49% respectively. *Panel d.* a summary of specific antigen binding by the scFvFc displayed on the lentivirus transduced cells. The table shows the percentage of transduced cells expressing X48-scFvFc-CD28-gp41 or PS11 scFvFc-CD28-gp41 that bind to their cognate or irrelevant biotinylated antigens as visualized by APC staining and their corresponding MFI values.

To establish conditions for mammalian cell display of scFvFc antibodies, 293T cells were transduced with PS11-scFvFc-CD28-gp41-IRES-ZsGreen encoding lentiviruses. As shown in Figure 5a, transduced cells efficiently expressed both PS11-scFvFc, as detected by APC-anti-human Fc and ZsGreen. The difference in transgene expression could be a result of either higher sensitivity of APC-anti-human Fc staining and/or less efficient CAP-independent IRES driven expression of ZsGreen. Importantly, cell-surface expression of scFvFc was detectable at very low multiplicity of infection. Quantification analysis revealed that at MOI of 1, there was about 5000–8000 of PS11-scFvFc-CD28-gp41 surface expressed molecules per transduced human cell (see Material and Methods for details on quantification methods).

Antigen binding specificity of surface expressed scFvFcs were confirmed by incubating cells transduced with CD28-gp41-IRES-ZsGreen lentiviruses encoding either 11A-, PS11- or X48-scFvFc with biotinylated antigens, followed by APC-streptavidin staining. As shown in Figure 5, upon incubation with its specific biotinylated GD03-Fc protein antigen, 11A-scFvFc expressing cells could be easily detected (Panel c), while the control PS11-scFvFc expressing cells exhibited only background levels of APC-streptavidin staining (Panel b). Similarly, following incubation with a biotinylated N-terminal CXCR4 peptide, 37% of cells transduced with the X48-scFvFc-CD28-gp41-IRES-ZsGreen lentivirus stained positive with streptavidin-APC (Figure 5d). In contrast, only background staining was detected when the same transduced cells were incubated with biotinylated GD03-Fc protein. Comparable results were seen with PS11-scFvFc, which specifically binds TRM peptide but not an irrelevant GD03-Fc protein.

### Selection and enrichment of rare scFvFc antibodies displayed on the surface of lentivirus transduced human cells

To determine if transduced cells expressing scFvFc fusion proteins on their surface could serve as a platform for isolating new scFvs, 11A-scFvFc- scFvFc-CD28-gp41 and PS11-scFvFc-CD28-gp41 cells were mixed at decreasing concentrations of the former and the sensitivity of the isolation and enrichment process was evaluated. Our initial results indicated that, at one-week post-viral transduction, a single round of selection by direct FACS sorting of high antigen binding/ZsGreen expressing cells, resulted in a three log enrichment of antigen specific 11A-scFvFc surface displayed cells, from a background cell population. However, isolation of 11A-scFvFc expressing cells could not be reliably achieved at a mixing ratio below 1∶1000 (data not shown).

**Figure 6:**
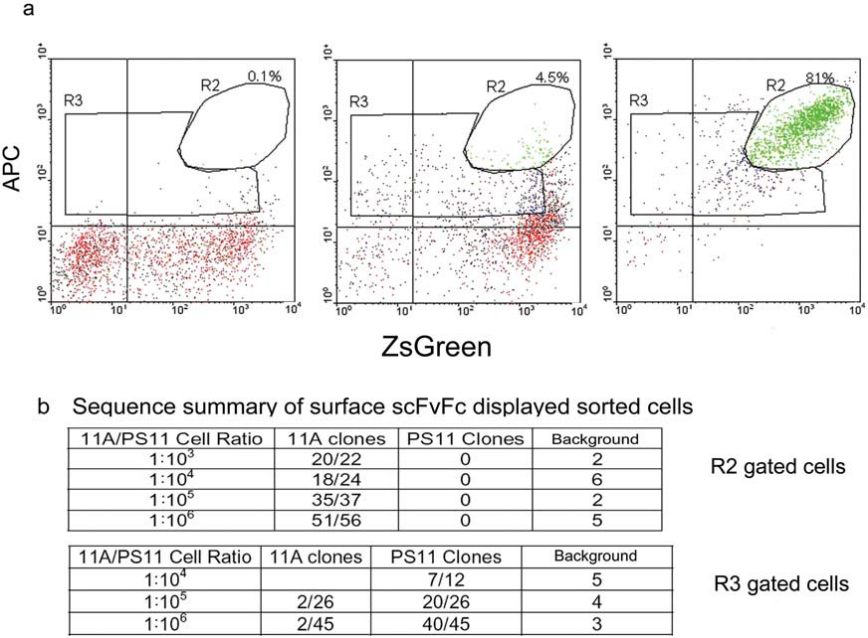
Selection of rare scFvFc expressing cells by a two-step magnetic bead and FACS sorting procedure. 293T cells were transduced with either PS11-scFvFc-CD28-gp41-IRES-ZsGreen or 11A-scFvFc-CD28-gp41-IRES-ZsGreen encoding lentiviruses. These two transduced cell populations were mixed at different 11A- to PS11-scFvFc ratios with a total cell number of ∼10^9^. Mixed cells were incubated with the biotinylated GD03-Fc protein antigen and APC-streptavidin. APC-positive cells were subjected to an enrichment using anti-APC magnetic micro-beads followed by FACS sorting. Cells from either R2 gate (high APC expressing cells) or R3 gated (low APC expressing cells) were isolated, propagated, re-stained for biotin-GD03-Fc binding and also analyzed for their scFv gene content by PCR rescue and DNA sequencing. *Panel a*, the FACS dot-blot profiles of APC- and ZsGreen positive cells within the 11A∶PS11 scFvFc expressing cell population (initial mixing ratio at 1∶10^6^), before magnetic beads enrichment (left panel), following magnetic bead enrichment (middle panel), and the R2 gated cells after sorting and expansion (right panel). *Panel b*, DNA sequencing results of individual clones recovered from the R2 or R3 gated pools of cells sorted from different 11A∶PS11 scFvFc expressing cell ratios (1∶10^3^–1∶10^6^). Note that the irrelevant sequencing data are most likely originated from cloning background.

We hence modified the selection procedure in order to improve sensitivity for specific antibody detection. Lentivirus transduced cells were pre-sorted for ZsGreen expression soon after transduction. Upon further propagation, 11A- and PS11-scFvFc cells were mixed at different ratios; incubated with a fixed concentration of biotin-GD03-Fc protein and streptavidin-APC; and an enrichment step, using MACS-anti-APC microbeads (Miltenyi), was performed prior to FACS analysis and sorting of streptavidin-APC positive cells. FACS analysis showed that the enrichment procedure was highly efficient at cell mixing ratio of 1∶10^6^, reaching at least 45-fold (compare Figure 6a R2 gates of left and middle panels; note 0.1% cells within R2 gate are non-distinguishable from a background value). Following magnetic bead enrichment, high (R2 gate) and low (R3 gate) APC-stained and ZsGreen expressing cells were again sorted and the two cell populations (about 500–1000 cells) were propagated for one week. A portion of cells was further propagated to reach a sufficient number for re-staining with biotinylated GD03-Fc protein and APC-conjugated streptavidin, while scFv genes from the remaining cells were rescued by PCR amplification of genomic DNA for rapid recloning and scFv DNA sequence analysis. As shown in Figure 6a (right panel), upon propagation and re-staining, the majority of originally high APC staining cells were positive for GD03-Fc binding (81% within the R2 gate), while cells isolated from R3-gate expressed low levels of ZsGreen and human Fc staining, but did not bind to biotin-GDO3-Fc (data not shown). DNA analysis confirmed that 51/56 of the clones generated from the R2-gated cells were positive for the 11A scFvFc gene (Figure 6b). In contrast, 40/45 clones from the R3-gated cells encoded the PS11 scFvFc gene and only 2/45 clones expressed the 11A scFvFc. Overall, magnetic beads enrichment combined with FACS sorting of high antigen binding/ZsGreen expressing cells resulted in a 10^6^ fold-enrichment of antigen binding cells in a tandem two-step round of selection.

### Neutralization of infection mediated by cell-surface displayed scFvFc

**Figure 7:**
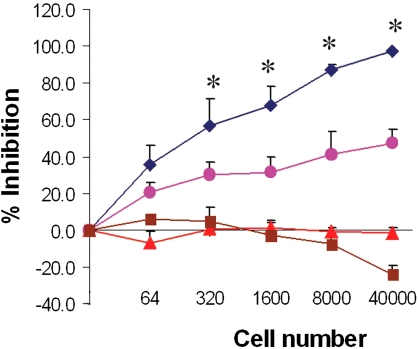
Neutralization of SARS-CoV TOR2 spike protein pseudotyped lentiviral infection of ACE2 expressing cells mediated by cell-surface displayed anti-TOR2 spike 80R-scFvFC antibodies. Single round, TOR2 spike protein pseudotyped luciferase expressing lentiviral particles were incubated with increasing concentrations of 293T cells expressing on their surface the 80R scFvFc (blue diamond) or the control PS11 scFvFc (pink circle). As a control for non-specific reporter virus absorption, a VSV-G pseudotyped luciferase reporter lentivirus was also incubated with 80R scFvFc (red triangle) or PS11 scFvFc expressing cells (brown square). Following incubation, the supernatant containing remaining lentivirus was used to infect permissive cells that express the ACE2 receptor for SARS CoV. At 48 hours post transduction, cells were harvested, luciferase activity was measured and relative inhibition of reporter virus infection was calculated. Asterisks in the designated points represent a statistical analysis that was performed to verify significant differences in % of inhibition between viral absorptions with the 80R- or PS11-scFvFc at a specific cell number point (P<0.05).

It was next determined if transduced human cells expressing the unique surface anchored scFvFc could be used directly in a biological screen. The anti-SARS-CoV 80R antibody was chosen as a model system for these studies [19], [23]. Luciferase expressing lentivirus pseudotyped with the cognate Tor2 Spike protein was absorbed with increasing numbers of 80R-scFvFc or irrelevant PS11-scFvFc expressing cells, prior to their single round infection of permissive cells expressing the SARS-CoV receptor, ACE-2. Non-specific virus absorption by scFvFc expressing 293T cells was also controlled with VSV-G pseudotyped viral particles. As shown in Figure 7, close to 100% neutralization of TOR2 pseudotyped virus infection was achieved following incubation with 80R-scFvFc surface expressing cells as compared with 40% inhibition seen by incubation with the PS11 scFvFc expressing cells. In contrast, neither PS11 nor 80R scFvFcs could neutralize the infection of permissive cells by VSV-G pseudotyped particles. Thus, very few numbers of transduced human cells expressing a unique scFvFc can be used directly in biological screens with exquisite sensitivity.

## Discussion

This study demonstrates that human cells, the natural host of human antibodies, can serve as a scaffold for antibody surface expression, screening and isolation using lentivirus display. Several thousand functional, bivalent scFvFc fusion proteins were stably expressed on the surface of human cells. The scFvFc antibodies when fused to the CD28/CD8 transmembrane moieties were evenly distributed on the cell surface. A number of technical features of this lentiviral display system were explored in this study and deserve further comment.

First, transduction of human cells with a self-inactivating, bicistronic lentiviral vector encoding scFvFc-CD28-gp41 and ZsGreen proteins, combined with magnetic beads enrichment and FACS sorting, resulted in a 10^6^-fold enrichment of specific antibody expressing cells in one single tandem, two-step procedure, to levels comparable or superior to those achieved by other microbial display systems [24], [25]. Together with optimized scFv PCR rescue and re-expression, this system should allow the development of rapid, iterative antibody enrichment procedures. Indeed, in the scFv gene PCR rescue experiment described in Figure 6b, only one week of cell propagation was used and shorter times are clearly possible. It should be noted that two cell populations were commonly detected as low (R3 gate) and high (R2 gate) ZsGreen expression and GD03-Fc antigen binding. This occurred despite optimized amounts of biotinylated GD03-Fc protein that were used to limit APC-streptavidin cross-reactive staining of irrelevant PS11 scFvFc transduced cells (Figure 5b). Sequencing analysis confirmed that the majority of the R3-gated cells expressed the irrelevant PS11 scFvFc, while the vast majority of the R2-gated cells encoded 11A scFvFc (Figure 6b). Thus, careful consideration of the background threshold for each antigen, and sorting for only high antigen binding and ZsGreen expressing cells, are useful guidelines to maximize the recovery of antigen binding positive cells. In addition, the positive signal to noise MFI ratio could potentially be increased through adjusting levels of biotin conjugation to an antigen probe or through an extra biotin/avidin amplification step. Initial MOI for transductions should also be controlled to ensure both library diversity and antibody expression.

Second, although not explored in detail in this study, human B-cells are also efficiently transduced and display high levels of scFvFc-CD28-gp41 antibody on their surface (data not shown). The endogenous biochemical pathways that are responsible for hypermutation of antibodies are constitutively expressed in human B-cells [11], [12] and/or can be further manipulated in an inducible manner through retroviral gene transfer [26], [27]. Thus, affinity maturation by somatic hypermutation of displayed antibodies may be possible and would have the advantage of occurring in stable transduced cell lines, allowing easy subcloning and recovering of the scFv gene.

Third, the lentiviral display system should complement existing antibody display technologies by virtue of providing properties unique to antibody expression in human cells. For example, surface display of human antibodies modified by CDR tyrosine-sulfation has not been reported for other antibody display systems. Based on the fact that V-region tyrosine sulfation did occur on individual surface displayed scFvs (Figure 3c), expression of scFvFc proteins on human cells should allow isolation of antibodies exhibiting unique properties of functional tyrosine sulfation that could otherwise be missed through expression by ribosomal display or other microbial display systems, where post-translational sulfation of tyrosine residues by Golgi associated tyrosine-O-sulfonyl transferases does not occur [28]. In addition, the antibodies isolated *via* human cell screening should express efficiently in mammalian cell systems, without the unpredictable problems that are frequently seen in expression of antibodies selected by phage display.

A fourth distinct advantage of this system is that the scFvFc displaying human cells could be used in direct biological screens with remarkable sensitivity. Studies described in Figure 7 showed that virus neutralization activity could be detected with as few as 320 scFvFc expressing cells. Although not yet tested, it also seems likely that transduced cells expressing bivalent surface displayed antibodies may mediate cross-linking of antigen molecules expressed on the surface of human target cells following co-incubation, which could lead to positive or negative modulation of signaling and biological responses of the target cells. This may provide an early and direct screen to interrogate the desired biological activity of the antibodies without the need to initiate costly soluble antibody production and purification procedures until the lead antibodies are identified.

Finally, an important feature of this system is that the functional scFvFc antibodies were successfully pseudotyped and expressed on lentiviral surface (Figure 4). Thus, the scFvFc pseudotyped lentiviral particles could also serve as a highly specific targeted gene delivery vehicle, particularly when fusion functions are provided *in trans*, as has been recently reported [29], [30].

In summary, relative ease in generating high titer lentiviral stocks [14], [31]–[33] combined with the high permissiveness of 293T cells to lentiviral transduction provides a platform which, we believe, could be easily scaled up to host a large diverse human scFvFc library. A human scFvFc-display master cell bank would serve as a rich source for screening and isolation of high affinity human scFv. By fully exploiting this lentivirus antibody display system, the isolation of new human antibodies with unique structural and biochemical properties complementing existing display systems should be possible. Transfer of large and diverse human scFv libraries from phage to the lentivirus mediated scFvFc cell surface display platform and panning against common target antigens using these alternative screening systems are ongoing. These comparative studies, as have been similarly performed for yeast and phage [34], will help define the value of lentivirus display in the discovery of novel therapeutic human mAbs.

## Materials and Methods

### Construction of mammalian cell surface display

All scFv antibodies used in this study were originally derived from the Mehta I/II non-immune human scFv-phage libraries [18]. ScFv were cloned into a pcDNA 3.1-based expression vector as an Sfi-I/Not-I 856 bp insert, and fused in frame with a human Fc-region (hinge-CH2-CH3) that had been amplified by PCR and cloned into pCDNA 3.1 as a Not-I/Xba-I fragment. Transmembrane (TM) anchoring moieties were amplified by PCR, using the appropriate primers and templates (sequences available upon request) and were cloned, in-frame, as Xba-I/Pac-I digested PCR fragments, into the pCDNA 3.1-PS11-scFvFc expression vector. A C9 sequence [34] was inserted N-terminus of all TM domains. Plasmids DNA were sequenced and verified for cell-expression.

### FACS analysis of functional expression of scFvFc on the surface of transiently transfected cells

293T cells were seeded a day before transfection on a 6 well plate. At the day of transfection, the 95% confluent cells were co-transfected with 4 µg of each of the plasmids DNA (see Figure 1) and 0.1 µg of pCDNA3.1-CMV-GFP, using lipofectamine 2000 (Invitrogen). APC-conjugated anti-human Fc antibody (Jackson ImmunoResearch) was used to determine cell-surface expression level of scFvFc. 5×10^5^ 293T cells were harvested using 5 mM EDTA at 48 hour post transfection, washed with PBS, and incubated on ice for 1 hour with a PBS staining solution containing 1 µl of APC-conjugated anti-human Fc antibody (Jackson ImmunoResearch) and 2% BSA per sample. Following incubation, cells were washed 3 times with PBS+2% BSA and analyzed by FACS. Mock- transfected cells incubated with the APC-conjugated anti-human Fc antibody were used as controls. Transfection efficiency of each sample was verified through GFP expression and analyzed concurrently by FACS.

To analyze if cell-surface expressed PS11-scFvFc proteins remain functional for specific antigen binding and whether the different TM domains have an effect on scFvFc function, 293T cells were transiently transfected and harvested as described above followed by incubation with biotinylated Tat Recognition Motif (TRM) peptide (“Macromolecular Resources”, CO) at 10 µM final concentration/sample on ice for 30 minutes and washed 3× with PBS+2% BSA. Cells were further incubated on ice, in the dark, with a staining PBS solution containing 2% BSA and 1 µl of streptavidin-APC (Jackson ImmunoResearch). Finally, cells were washed 3 times with PBS+2% BSA and analyzed for APC staining by FACS. GFP expression of transfected cells was also analyzed to standardize transfection efficiency.

### Confocal microscopy analysis of scFvFc cellular localization in transiently transfected 293T cells

293T cells were transfected with DNA encoding for a PS11-scFvFc-CD28-gp41-IRES-ZsGreen, PS11-scFvFc-gp41-IRES-ZsGreen or CMV-ZsGreen plasmids. The latter served as a negative control for cells that do not express scFvFc on their surface. At 48 hours post transfection, samples were fixed with 3.9% paraformaldehyde (SIGMA) for 30 minutes and washed once with PBS. Subsequently, cells were incubated with PBS/0.1 M glycine (SIGMA) for 10 minutes, followed by washing once with PBS and permeabilization with PBS/0.05% saponin (SIGMA) for additional 30 minutes. Upon further washing, cells were blocked with PBS supplemented with 2% BSA and PBS/0.05% saponin for 30 minutes and incubated in the dark with an anti-human Fc-rhodamine antibody (Jackson ImmunoResearch) for 1 hour. Finally, cells were washed and mounted for flurescence microscopy by using ProLong antifade kit (Invitrogen). Images were acquired by using BIO-Rad Radiance 2000 laser scanning confocal microscope using a Nikon 60× camera.

### Metabolically radioisotope labeling and immunoprecipitation of scFvFc-CD28-gp41 fusion proteins

Surface displayed scFvFc post-translational sulfation analysis was performed as described earlier [17]. Briefly, 293T cells were transiently transfected with pCDNA3.1-scFvFc-CD28-gp41 expression plasmids using lipofectamine 2000 (Invitrogen). Eighteen hours later, cells were washed twice with PBS. To determine protein sulfation, one set of cells was incubated with sulfate-free media (Sigma), supplemented with 500 µCi of [^35^S]-sulfate (PerkinElmer), with or without 100 mM sodium chlorate. For analyzing protein expression, another set of cells was incubated in parallel with L-Methionine and L-cysteine free DMEM medium (GIBCO), supplemented with 200 µCi of [^35^S]-labeled cysteine-methionine mixture (PerkinElmer), with or without 100 mM sodium chlorate. Cells were collected 24 hours later and lysed with solubilization buffer containing 100 mM (NH_4_)_2_SO_4_, 20 mM Tris (pH 7.5), 20% glycerol, and 1% 3-[(-cholamidopropyl) dimethylammonio]-2-hydroxyl-1-propanesulfonic acid (CHAPSO, Anatrace, Maumee, Ohio) in the presence of 1× complete protease inhibitor mixture (Roche Molecular Biochemicals, Indianapolis, Ind.). Cell lysates were incubated overnight at 4°C with protein A sepharose beads (GE Healthcare) and washed three times with PBS containing 0.1%Tween 20. Finally, proteins were eluted from the beads by 2XSDS buffer separated by 12% SDS-PAGE, and visualized by autoradiogram.

### Generation of scFvFc-TM/VSV-G pseudotyped lentiviruses and 293T cells stably expressing scFvFc-TM through lentiviral transduction

A self-inactivating pHAGE lentivector (a gift from R. Mulligan) was modified to accommodate subcloning of Sfi-I/Pac-I scFvFc-TM DNA fragments. pHAGE lentivector has a CMV promoter that drives the expression of the transgene. It also expresses the Zoanthus Green Fluorescent protein (ZsGreen) gene *via* an IRES sequence, which can be used to monitor and normalize transduction efficiencies.

For the production of VSV-G pseudotyped lentiviral particles, total of 5×10^6^ 293T cells were seeded on 100 mm diameter plates, and co-transfected the next day with 10 µg of the pHAGE lentivector and packaging plasmids (10 µg HIV-1 Gag-Pol, 1 µg pCMV-Rev1b, and 2.5 µg pCMV-VSV-G), using the Ca-Phosphate method. At 48 and 72 hours post transfection, supernatant was collected and cleared by centrifugation (2100 rpm; 15 minutes) and sterile filtered through a 0.45 µm filter. Viral supernatant was concentrated by ultracentrifugation (21,000 rpm; 2 hours) through a 20% sucrose cushion, aliquoted and stored at −80°C. The titer of the pseudotyped virus particles was evaluated either by RT assay or by transduction of HeLa cells with increasing dilutions of the lentivirus stock and measurement of ZsGreen marked cells or APC-anti-human Fc staining by FACS analysis. Importantly, incorporation of the scFvFc-CD28/CD28-gp41 fusion protein did not lower the titer of viral particles (data not shown).

293T cells were transduced, in the presence of 8 µg/ml polybrene, with above generated recombinant lentiviruses at different MOI as indicated for 4 hours. Functional scFvFc expression on the transduced cell surface was analyzed by FACS, following a similar protocol as described above, first at 48 hours post-trandsuction then again after longer periods of propagation to confirm stable scFvFc expression. Biotinylated peptide or protein antigens and their concentration used for specific antibody binding analysis are described in the figure legends.

### Incorporation of functional scFvFc-TM proteins into lentivirus particles

#### Viral capture assay

A 96-well plate was coated with different antigens overnight at 4°C, using a coating buffer-NaCO3/HCO3 (pH 9.6). The following antigens were used: commercial biotinylated TRM peptide 50 µM, biotinylated GD03 (S1-RBD)-Fc 20 µg/ml, or 20 µg/ml BSA. Duplicated wells were blocked with 1% BSA at 4°C for 1.5 hour prior to incubation with equal amounts of concentrated lentiviral particles (based on reverse transcription activity) expressing on their surface either the 11A or PS11 scFvFc antibodies for additional 1.5 hour at 4°C. Wells were then washed 5 times with 200 µl PBS and reverse transcription analysis was performed on eluted particles.

#### Western blot analysis

Equal amounts (based on p24 antigen levels) of concentrated lentiviruses were lysed using RIPA lysis buffer (50 mM Tris-HCl pH 7.4; 150 mM NaCl; 1 mM PMSF; 1 mM EDTA; 1% NP-40; 1% sodium deoxycholate; 0.1% SDS) supplemented with protease inhibitors (Roche, Indianapolis, Ind.) and resolved by SDS-PAGE under reducing conditions. Upon transfer, nitrocellulose membranes were blocked with 5% skim milk and proteins were probed with a mouse anti HIV-1-p24-HRP antibody (Immuno-Diagnostics), or a goat anti-human Fc-HRP antibody (Pierce) followed by detection using an enhanced chemiluminescence kit (Amersham).

#### Immunofluorescence of pseudotyped viruses

Hela cells grown overnight on 12 mm cover-slips were incubated at 4°C for 30 minutes with media containing 10 mM HEPES, pH-8.0, followed by incubation for additional 2 hours with VSV-G pseudotyped viral particles expressing surface PS11-scFvFc-CD28-gp41-ZsGreen, or control ZsGreen virus. Samples were fixed with 3.9% paraformaldehyde (SIGMA) for 30 minutes. Cells were washed once more with PBS and incubated with PBS/0.1 M glycine (SIGMA) for 10 minutes, followed by another wash in PBS and permeabilization with PBS 0.05% saponin (SIGMA) for 30 minutes. Samples were then blocked with PBS supplemented with 2% BSA and 0.05% saponin for 30 minutes and incubated with either a mouse anti-p24 antibody (AG3.0; NIH AIDS Research and Reference Reagent Program) or with an anti-human Fc-rhodamine antibody (Jackson ImmunoResearch) for 1 hour. Cells were then washed and incubated with Cy2-labeled anti-mouse IgG antibody (Jackson ImmunoResearch) for an additional hour. Controls include virus-bound cells incubated with the secondary antibody alone. Finally, samples were mounted for fluorescence microscopy by using ProLong antifade kit (Invitrogen). Images were acquired by using BIO-Rad Radiance 2000 laser scanning confocal microscope using a Nikon 60×.

### ScFvFc quantification on the surface of transfected or transduced cells

To quantify the number of scFv-Fc molecules on the surface of transiently transfected or lentivirus transduced cells, the “Quantum Simply cellular anti-mouse IgG kit” was used (Bangs Laboratories, Inc.). Briefly, cells expressing on their surface the scFvFc with a C9 tag were incubated with 10 µg of an APC conjugated mouse anti-C9 monoclonal antibody 1D4 (Invitrogen-Molecular Probes) for 1 hour on ice in PBS supplemented with 2% BSA. Calibration beads with known binding capacity of mouse IgG molecules on their surface were treated with the same conditions as the cells. Upon washing, both cells and calibration beads were analyzed for APC staining intensity by FACS. Number of scFv-Fc molecules on cell surface was determined by plotting a calibration curve, using the QuickCal quantitative software from Bangs labs.

### Isolation and enrichment of rare 11A scFvFc expressing cells

#### Transduction, ZsGreen sorting, and antigen staining of scFvFc expressing cells

As a model for the isolation of rare antibody from a population of scFvFc expressing cells, 293T cells were transduced with lentiviruses encoding either 11A scFvFc or PS11 scFvFc at MOI of one. The transduced cells were propagated for one week to ensure stable expression and then sorted based on their ZsGreen expression. Upon further propagation, ZsGreen sorted cells were counted and mixed at the depicted ratio of 11A/PS11 scFvFc. For the detection and isolation of 11A scFvFc transduced cells, total mixed cells were blocked with 2% BSA for 30 minutes followed by staining with biotinylated GD03-Fc at an optimized concentration of 2.65 µg/ml (GD03-Fc protein was biotinylated using the EZ-link NHS-biotin, PIERCE) for 30 minutes on ice. Cells were then washed 3 times with PBS and stained with streptavidin-APC in PBS/2% BSA.

#### Magnetic beads enrichment and FACS sorting of APC-positive 11A scFvFc expressing cells

Following staining with APC-Streptavidin, cells were washed and re-suspended in 500 µl PBS buffer containing 0.5% BSA and 2 mM EDTA. Cells were then labeled with MACS-anti-APC magnetic beads (20 µl/10^7^ cells, Miltenyi) for 10 minutes at room temperature. Single-cell suspension was loaded on pre-washed MS magnetic columns that were placed in a magnetic field. Upon removing unbound cells through 3 times of washing, APC labeled cells were recovered by removing the columns from the magnetic field and plunging the cells using 1 ml of the above buffer. Cells isolated by anti-APC magnetic beads were analyzed by FACS. Two populations of APC positive cells, R2 and R3, were sorted. Upon propagation for one week, APC positive R2 and R3 sorted cells were divided into two portions. One part was subjected to PCR isolation of scFvFc fragments. Amplified scFvFc fragments were cloned into the TOPO TA cloning vector (Invitrogen) followed by DNA sequencing. Rest of the cells were further propagated for 1–2 weeks to achieve adequate numbers for a repeated staining of cell-surface scFvFc by biotinilated GD03-Fc and APC-Streptavidin.

### Inhibition of SARS-CoV spike protein pseudotyped lentiviral infection by cell-surface displayed 80R scFvFc

SARS-CoV spike protein (TOR2 strain) pseudotyped lentiviruses, expressing a luciferase reporter gene, were incubated at 4°C for 30 minutes with increasing concentrations of 293T cells expressing on their surface the anti-TOR2 spike 80R-scFvFc or with cells expressing PS11-scFvFc on their surface as a control. Both 80R- and PS11-scFvFc surface-expressing cells were also incubated with VSV-G pseudotyped viral particles for the analysis of non-specific viral absorption. Following absorption, viral supernatant was used to infect 293T cells expressing the ACE2 receptor as described [23]. Neutralization of infection was determined by measuring the luciferase activity in the target cells following transduction, using EG&G Berthold Microplate Luminometer.
